# Preparation of Patchouli Oil Microemulsion Gel and Its Topical Application to Ameliorate Atopic Dermatitis in Mice

**DOI:** 10.3390/gels10120796

**Published:** 2024-12-05

**Authors:** Tingting Chen, Changjin Xu, Min Wang, Yan Cui, Riqing Cheng, Wenyao Zhang, Xin Gao, Laibing Wang, Herima Qi, Shuyan Yu, Jianping Chen, Lan Ma, Huiqing Guo

**Affiliations:** 1 School of Pharmacy, Inner Mongolia Medical University, Hohhot 010100, China; chen@stu.immu.edu.cn (T.C.); changjin.xu@immu.edu.cn (C.X.); 20100003@immu.edu.cn (M.W.); rqcheng@immu.edu.cn (R.C.); zwy1375123@stu.immu.edu.cn (W.Z.); gao0804@immu.edu.cn (X.G.); lbwang1966@163.com (L.W.); qiherima@immu.edu.cn (H.Q.); lee_2014@163.com (S.Y.); 20100001@immu.edu.cn (L.M.); 2 College of Humanities Education, Inner Mongolia Medical University, Hohhot 010100, China; 20060159@immu.edu.cn

**Keywords:** patchouli oil, microemulsion, microemulsion gel, preparation process, quality evaluation, atopic dermatitis

## Abstract

Patchouli oil (PO) is a natural substance famous for its immune-enhancing and anti-inflammatory effects. Atopic dermatitis (AD) is characterized by epidermal gene mutations, skin barrier dysfunction, and immune dysregulation, making patchouli volatile oil a potential candidate for AD treatment. Initially, PO was mixed with ethyl oleate (EO), castor oil ethoxylated ether-40 (EL-40), anhydrous ethanol, and water to form a patchouli oil microemulsion (PO-ME) system. The formulation ratios were optimized using the Box–Behnken design-effect surface method, and their products were characterized for type, particle size, polydispersity index (PDI), and appearance. Additionally, patchouli oil microemulsion gel (PO-MEG) was developed with a specified concentration of 1.5% carbomer-940 as the matrix, and its pH, stability, viscosity, and permeability were evaluated. We assessed the irritation tests of PO-MEG using a rat self-control model and the Cell Counting Kit-8 (CCK-8) assay. The results demonstrated that should be attributed to non-irritating. This study also assessed the efficacy of optimized PO-MEG on AD-like symptoms using a 2,4-dinitrochlorobenzene (DNCB)-induced BALB/c mouse model. Compared with the model group, the in vivo efficacy studies have shown the PO-MEG group significantly reduces dermatitis scores, mast cell counts, epidermal thickness, and levels of pro-inflammatory cytokines and immune factors in skin homogenates. This suggests that PO-MEG would become a safer topical formulation for treating atopic dermatitis.

## 1. Introduction

Atopic dermatitis (AD) is a common chronic inflammatory skin disease, increasing worldwide year by year [1,2]. The etiology of AD is extremely complex, primarily resulting from genetic and environmental factors. Its pathophysiological mechanisms include epidermal gene mutations, skin barrier dysfunction, and immune dysregulation [3,4], while the impairment of the skin barrier is very crucial for the development of AD [5]. The inflammatory pathogenesis of AD is primarily related to the activation of T cells and the infiltration of inflammatory cells into the skin [6]. T helper cells type 1 (Th1 cells) secrete interleukin 2 (IL-2) and interferon gamma (IFN-γ), while T helper cells type 2 (Th2 cells) secrete interleukin 4 (IL-4), interleukin 5 (IL-5), and interleukin 13 (IL-13). In acute AD, the Th2 cytokine IL-4 predominates, whereas IFN-γ plays a more significant role in chronic AD [7]. Additionally, the inflammation of AD can be characterized by elevated IgE levels.

At present, AD treatment relies on topical or oral glucocorticoids and calcineurin inhibitors [8], which are mainly suitable for moderate to severe patients while the local treatment options for mild to moderate patients are quite limited. The long-term or large-area use of topical glucocorticoids on the skin may lead to many adverse effects, including skin thinning, capillary dilation, pigmentation and disease rebound [9]. Additionally, it has been reported that calcineurin inhibitors can cause lymphoma, leukemia, and malignancy [10]. Therefore, there is a growing demand for innovative topical treatments that effectively reduce skin rash severity, alleviate itching, and minimize adverse effects.

Patchouli oil (PO), a natural herbal extract, exhibits various biological activities such as anticancer [11], anti-inflammatory [12], antiviral, antioxidant, and antimicrobial properties [13], as well as gastrointestinal tract protection [14]. Some researchers have confirmed that pogostone and patchouli alcohol in patchouli volatile oil possess antibacterial, anti-inflammatory, and immune-regulating effects, significantly inhibiting inflammatory cell infiltration [15]. Suo et al. [16] studied the anti-allergic effects of refined huodan through pharmacological experiments and found that refined huodan recipe possesses some obvious inhibitory effect on delayed-type hypersensitivity reaction of the auricular skin of mice induced by 2, 4-dinitrochlorobenzene. He et al. [17] conducted delayed-type hypersensitivity experiments and found that all doses of PO significantly inhibited the delayed-type hypersensitivity reaction in SD rats induced by 2, 4-dinitrochlorobenzene. Based on this, PO may be considered a candidate for treating atopic dermatitis (AD), which is inconsistent with its etiology [18].

This study aims to develop a new type of therapeutic drug for AD using patchouli oil as the primary agent and addressing clinical needs. However, due to the poor water solubility and unstable active ingredients of the volatile oil in patchouli, it is prone to loss and skin irritation, limiting its application range. As a new drug carrier, microemulsion (ME) is thermodynamically stable, and consists of an oil phase, water phase, emulsifier and co-emulsifier [19]. There are three types of MEs: O/W, W/O and bicontinuous phase. Among these, O/W type microemulsion can improve the bioavailability of drugs through local and systemic pathways, due to its strong solubilizing effect on drugs with poor water solubility [20]. Meanwhile, ME has emerged as a promising drug carrier due to its enhancing solubility, and bioavailability of insoluble drugs [21], improving transdermal permeability [22,23], and covering undesirable odors. However, traditional ME formulations suffer from poor adherence and short retention times upon local application, restricting their use in topical drug delivery [24]. The treatment of dermatitis being a local disease primarily requires the deposition and localization of drugs in the superficial layer of the skin [25]. Therefore, it is neither necessary nor a prerequisite for the drug to penetrate deeply into the skin.

To overcome these challenges, microemulsions can be formulated into a microemulsion gel (MEG). The gel matrix is beneficial to maintain skin moisture and restore the skin barrier [26]. Microemulsion gel exhibits uniformity and delicacy, offering good biocompatibility, stability [27,28], a non-greasy feel, easy wash-off, non-irritating properties, and efficient organism metabolism [29]. This suggests microemulsion gel is suitable for topical skin treatment and a promising carrier for volatile and water-insoluble drugs in transdermal drug delivery [30]. Microemulsion-based gels combine the advantages of both emulsion and gel, such as the attractive texture and excellent consistency. Moreover, it is easy to administer and ensure prolonged drug release [31].

## 2. Results and Discussion

### 2.1. Results of Methodological Examination

**Figure 1:**
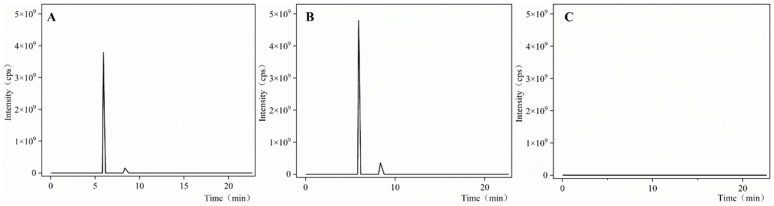
GC-MS chromatogram; (**A**) microemulsion sample, (**B**) reference substance, (**C**) blank solvent sample.

Peak areas (Y) for patchouli alcohol and pogostone were measured across concentration ranges of 35.175~562.8 μg/mL and 22.3~356.8 μg/mL, respectively. Linear regression provided the following equations: Y_1_ = 10^6^X + 5 × 10^6^ (R^2^ = 0.9999) for patchouli alcohol and Y_2_ = 906,900X – 2 × 10^7^ (R^2^ = 0.9991) for pogostone, demonstrating a strong linear correlation between concentration and peak area for both compounds. Precision analysis revealed relative standard deviations (RSDs) of 1.04% for patchouli alcohol and 1.7% for pogostone (n = 6), indicating high method precision. As shown in Figure 1, proprietary investigations validated the method’s efficacy in isolating target components and confirmed that the blank solvent did not interfere with the determination of patchouli alcohol and pogostone. Stability studies showed that RSDs for peak areas of patchouli alcohol and pogostone, measured at various time points, were 0.652% and 1.82%, respectively, demonstrating sample stability within 12 h. Method validation was supported by spiked recovery experiments, which yielded average recoveries of 95.36% (RSD = 2.99%, n = 6) for patchouli alcohol and 100.37% (RSD = 1.64%, n = 6) for pogostone, fulfilling the required criteria.

### 2.2. Pseudo-Ternary Phase Diagram Results

**Figure 2:**
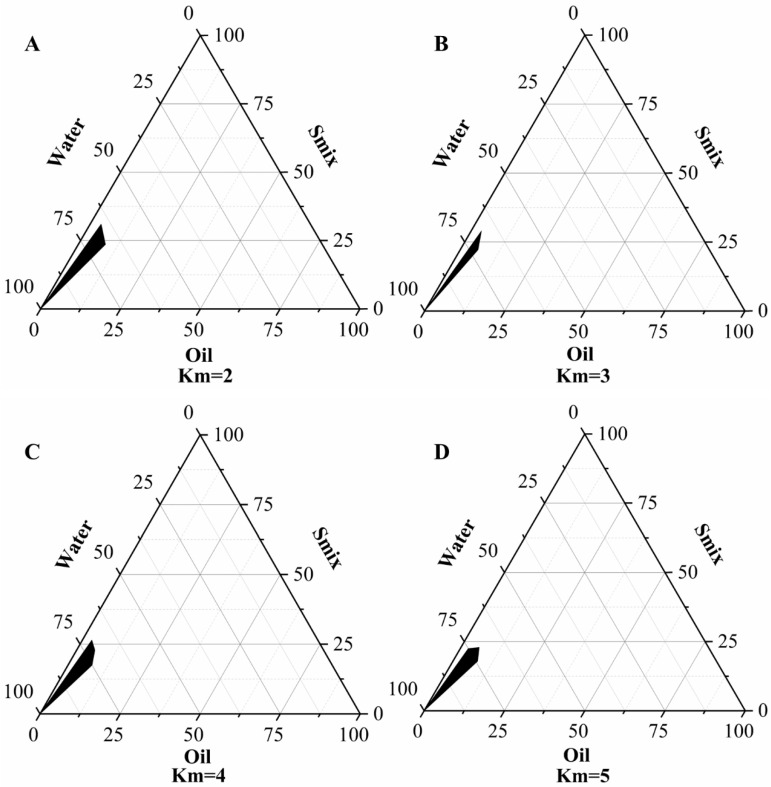
Pseudo-ternary phase diagram of the formed microemulsion for different Km’s; (**A**) Km = 2, (**B**) Km = 3, (**C**) Km = 4, (**D**) Km = 5.

The mass ratio of emulsifier to co-emulsifier is denoted as Km. With the change in the oil phase content and Km value, the size of the microemulsion region also varies. In the preparation of microemulsion, the interactions between the different proportions of the formulation components play a crucial role. As shown in Figure 2, these diagrams reveal that the oil phase dosage ranged from 2.5% to 8.7%, the emulsifier dosage varied between 14% and 20.69%, and the co-emulsifier dosage spanned from 3.5% to 10.35%.

### 2.3. Optimization Results of PO-ME Prescription

Table 1 displays the composition of the 17 experimental runs, with particle size (Y_1_) and the content of patchouli alcohol and pogostone (Y_2_, Y_3_) and comprehensive score (S) as response values.

**Figure 3:**
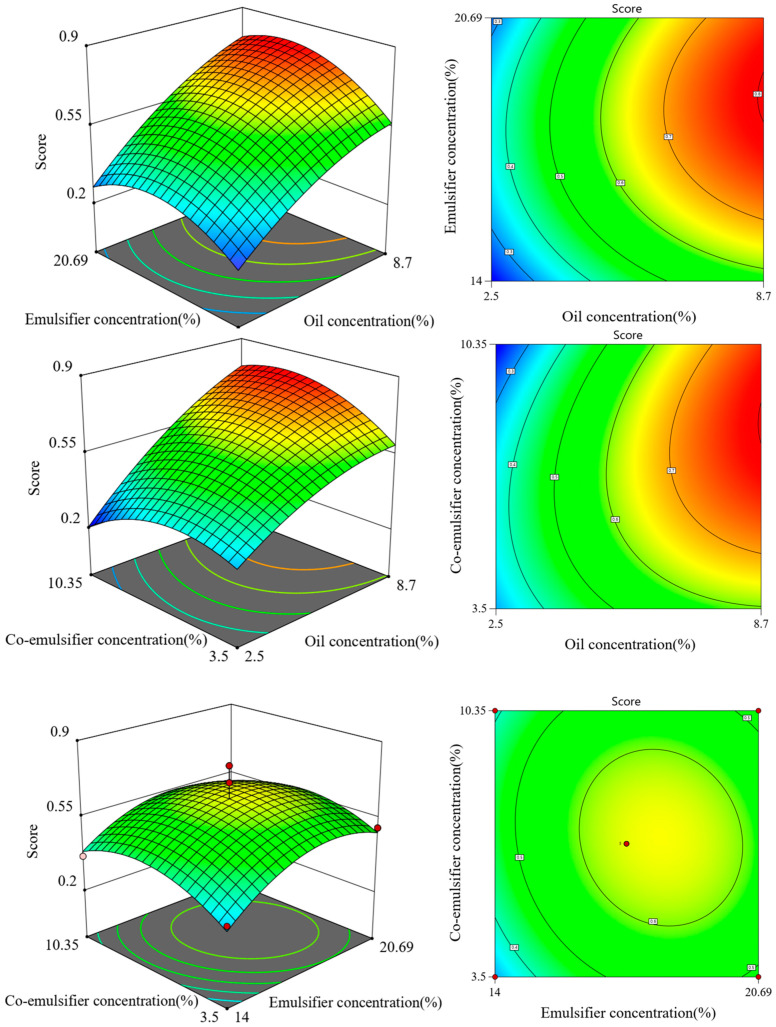
The 3D and 2D response surface plot.

Figure 3 depicts the effects of independent variables such as oil phase, emulsifier, and co-emulsifier on the overall score. The 2D contour plot shows the interactive effects of two factors. The experimental data were fitted to derive the following equation for the comprehensive score: S = 0.6373 + 0.2114A + 0.0636B + 0.0098C − 0.03286AB − 0.0962AC − 0.1532BC − 0.0272A^2^ − 0.2316B^2^ − 0.193C^2^, with an R^2^ value of 0.9086. The model’s F-value is 5.75, and the *p*-value of 0.0155 demonstrates statistical significance [32], indicating that the model provides a good fit and is suitable for analyzing the optimal formulation of the patchouli oil microemulsion. The factors’ influence on the preparation of the PO-ME were ranked in the following order: C > B > A. Analysis of the response surface and contour plots revealed that maintaining constant levels of emulsifiers or co-emulsifiers resulted in a decrease in comprehensive scores beyond certain thresholds, suggesting a non-linear relationship between these factors and the comprehensive score.

### 2.4. Validation of Experimental Results

Based on the model fitting results, the predicted values for particle size, Patchouli alcohol content, pogostone content, and the comprehensive score of the PO-ME from the optimal process were 24.82 nm, 36.76 mg/mL, 3.27 mg/mL, and 0.789, respectively. The adjusted formulation conditions were 8.7% ethyl oleate and patchouli oil, 19.6% EL-40, and 9.5% anhydrous ethanol. The actual measurements were 24.83 ± 0.12 nm (RSD = 0.04%) for particle size, 38.17 ± 0.31 mg/mL (RSD = 3.84%) for patchouli alcohol content, 3.23 ± 0.06 mg/mL (RSD = 1.22%) for pogostone content and a comprehensive score of 0.77 ± 0.01 (RSD = 2.41%). These results closely matched the predicted values from the regression equation, confirming the accuracy of the mathematical model. This validation supports the practical and theoretical feasibility of optimizing the preparation process of PO-ME using the response surface methodology.

### 2.5. Physicochemical Characterization Results of PO-ME

**Figure 4:**
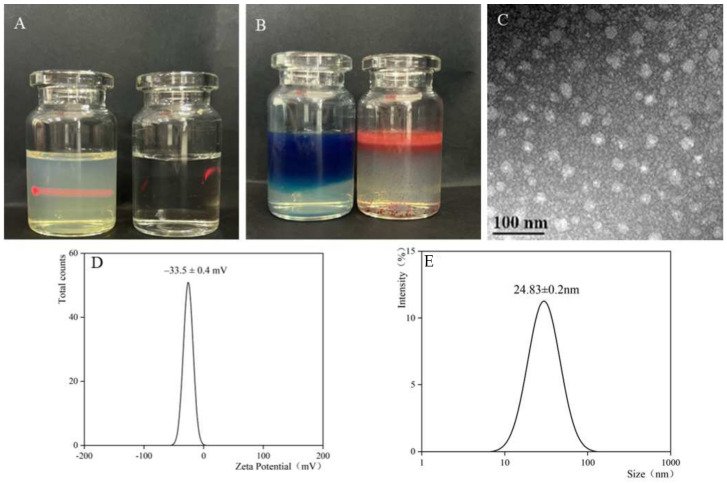
PO-ME quality evaluation diagram; (**A**) appearance, (**B**) type identification, (**C**) TEM images, (**D**) zeta potential, (**E**) particle size.

PO-ME is a light yellow, clarified transparent liquid that exhibits a Tyndall effect under laser irradiation in Figure 4A. The methylene blue diffusion rate of the microemulsion was notably higher than that of Sudan red III dye, confirming its oil-in-water (O/W) nature in Figure 4B. The microemulsion demonstrated a uniform particle size distribution with an average size of 24.83 ± 0.2 nm (n = 3) in Figure 4C,E. The average polydispersity index (PDI) was 0.174 ± 0.12 (n = 3), indicating a narrow size distribution. The average zeta potential was −33.5 ± 0.4 mV (n = 3) in Figure 4D, reflecting excellent stability. After six heating–cooling and freeze–thaw cycles, all three batches of the microemulsion remained clear and transparent, with a relative standard deviation (RSD) of 0.5 nm in particle size compared to the initial measurement, demonstrating strong thermodynamic stability. Additionally, after 30 min of high-speed centrifugation, the microemulsions remained homogeneous and transparent, with no delamination and a particle size RSD of 0.3 nm compared to pre-experiment measurements, indicating good centrifugal stability.

### 2.6. Physicochemical Characterization Results of PO- MEG

**Figure 5:**
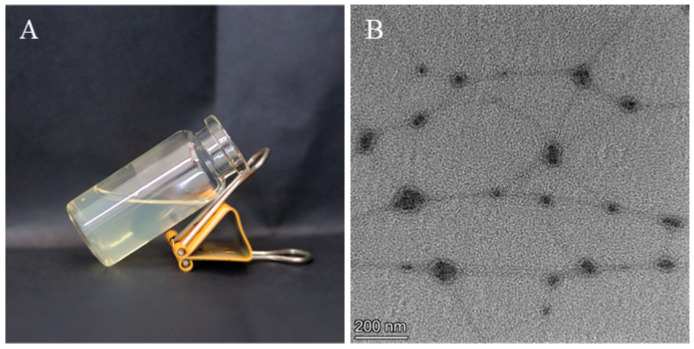
PO-MEG quality evaluation diagram; (**A**) appearance, (**B**) TEM images.

PO-MEG appeared as a light yellow, clarified, transparent, homogeneous, and fine semi-solid preparation, as depicted in Figure 5A. TEM revealed that PO-ME was contained within a reticulated structure, which limited free flow and contributed to the semi-solid nature of PO-MEG in Figure 5B. The pH, measured with a pH meter, was 6.08, falling within the acceptable range of 6–8 for topical gel agents. The viscosity, measured at 30 rpm using a viscometer, was 1628.43 ± 108.74 (n = 3) mPa·s. Three batches of PO-MEG stored at 40 ± 2 °C for 12 h exhibited a milky-white, opaque appearance with instability including delamination, thinning, and coarsening, indicating the need for storage at lower temperatures. After centrifugation, the three batches of MEG exhibited a light yellow, semi-transparent, homogeneous, and fine appearance, with no delamination or other unstable phenomena, indicating stability under high-speed centrifugation. Overall, these findings suggest that the PO-MEG remains stable under centrifugation and should be stored under cool conditions for optimal performance.

### 2.7. Results of Percutaneous Permeability Test

**Figure 6:**
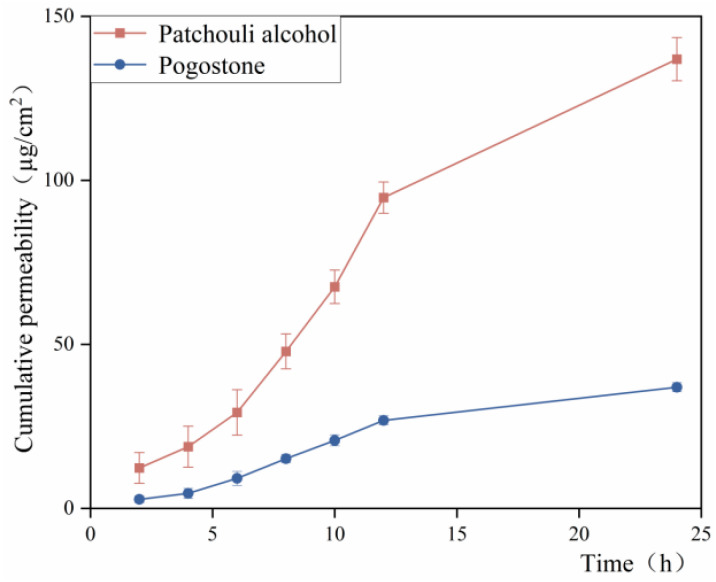
Percutaneous permeation curve of PO-MEG.

Figure 6 shows that the 24 h cumulative permeability of Patchouli alcohol and pogostone in the MEG group was 136.94 ± 6.58 µg/cm^2^ and 36.93 ± 1.4 µg/cm^2^, respectively in Table 2. The release of patchouli alcohol and pogostone from the PO-MEG occurred slowly over 24 h, with transdermal amounts stabilizing after 12 h. This behavior may be attributed to PO being retained in the deeper layers of the skin, allowing for more effective local action and indicating a slow-release effect of the gel. Additionally, the volatility of PO is mitigated by its formulation into the microemulsion gel, which helps to delay its evaporation and prolong the activity of its volatile components.

### 2.8. Results of Cytotoxicity Assay In Vitro

**Figure 7:**
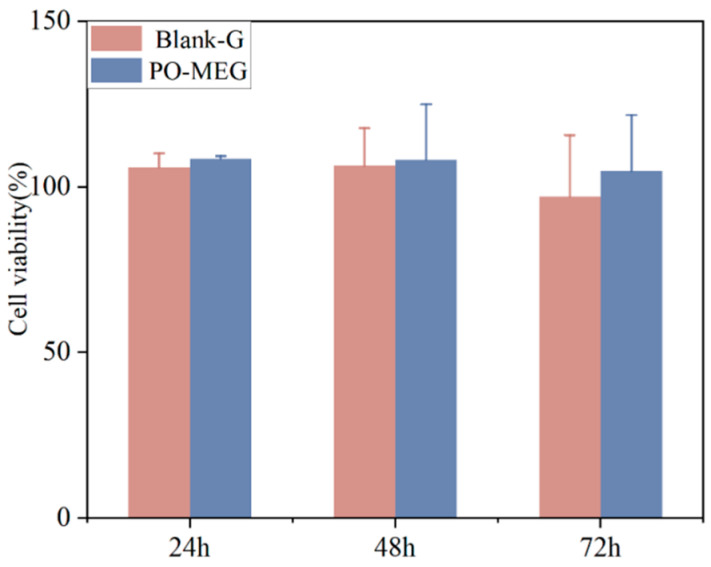
Cytotoxicity of different preparations on HaCaT cell (n = 3).

Cytotoxicity of PO-MEG and Blank-G was evaluated using the CCK-8 assay. As shown in Figure 7, treatment of HaCaT cells with Blank-G for 24, 48, and 72 h resulted in cell viabilities of 105.91%, 106.41%, and 97.03%, respectively. Similarly, treatment with PO-MEG for the same time points yielded cell viabilities of 108.54%, 108.21%, and 104.8%, respectively. The inhibition rates for both PO-MEG and Blank-G were less than 5%, indicating minimal cytotoxicity. These findings suggest that both PO-MEG and Blank-G are non-toxic to HaCaT cells and hold potential as candidates for skin drug-delivery systems.

### 2.9. Results of Skin Irritation Experiments

As shown in Table 3, after the application of the PO-MEG and Blank-G to the backs of rats, no significant redness or swelling was observed in either normal or compromised skin. This suggests that the PO-MEG possesses minimal skin irritation, indicating that it is very safe and non-irritated for skin administration.

### 2.10. Inhibitory Effect of Different Groups on DNCB-Induced Skin Damage in Mice

**Figure 8:**
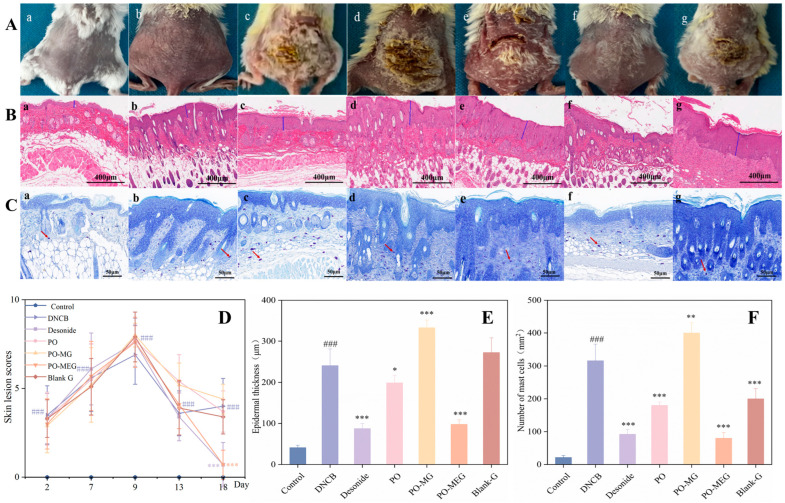
The skin damage images of mice in different groups in mice. (**A**) The skin injury of mice in each group on the 19 day; (**B**) H&E at 100× (scale bar = 400 μm); (**C**) toluidine blue at 400× (scale bar = 50 μm); (**D**) The skin damage scores (Means ± SD, n = 8); (**E**) Epidermal thickness of mice in different groups (Means ± SD, n = 4); (**F**) number of mast cells in different groups of mice (Means ± SD, n = 4). Compared with the control group, ### *p* < 0.001 and compared with the DNCB group, * *p* < 0.05, ** *p* < 0.01, *** *p* < 0.001.

The effects of different treatments on DNCB-induced skin damage were evaluated at 2, 7, 9, 13, and 18 days, and the severity of skin damage was scored accordingly in Figure 8A,D. Following three rounds of DNCB-induced skin damage, the damage reached its peak, with no significant differences observed in the degree of skin damage among the various groups of mice. After 10 days of treatment with various drugs, distinct therapeutic effects were observed. Topical application of PO-MEG and Desonide significantly reduced the severity of skin lesions compared to the DNCB group. The DNCB-treated group exhibited AD skin lesions characterized by hyperkeratosis, epidermal thickening, and an increased number of mast cells in the dermis, as illustrated in Figure 8B,E. Epidermal thickness and the number of mast cells were significantly elevated in the DNCB group compared to the control group in Figure 8C,F. In the PO-MG group, both epidermal thickness and mast cell count were notably higher than in the DNCB group, potentially due to irritation from the non-nanosized gel agent, which exacerbated the AD condition. Conversely, the PO, PO-MEG, and Desonide groups significantly reduced both mast cell numbers and epidermal thickness. The Blank-G group also significantly decreased mast cell count but did not reduce epidermal thickness, which may be attributed to irritation from the non-nanosized gel agent. This effect could be due to the gel matrix hydrating the stratum corneum, maintaining moisture at the application site, and thereby inhibiting mast cell proliferation.

### 2.11. Effects of Different Groups on DNCB-Induced Inflammatory Factors

**Figure 9:**
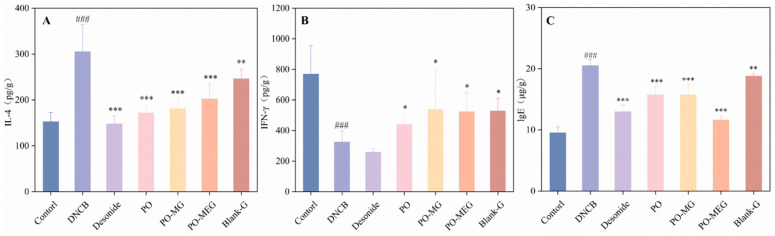
Effect of topical application of different drugs on IFN-γ, IL-4 and IgE levels in the skin of DNCB-induced AD mice (**A**) IFN-γ, (**B**) IL-4, (**C**) lg E. Compared with the control group, ### *p* < 0.001 and compared with the DNCB group, * *p* < 0.05, ** *p* < 0.01, *** *p* < 0.001.

As shown in Figure 9, the model group exhibited significantly elevated levels of IgE and IL-4 in the skin compared to the control group, while IFN-γ levels were significantly reduced. In contrast, the treatment groups demonstrated significant decreases in IgE and IL-4 levels, accompanied by marked increases in IFN-γ levels. Notably, the Dinocap group showed a significant reduction in IFN-γ levels. These findings suggest that all treatments, including PO preparations and the blank gel, effectively lower IgE and IL-4 levels in the skin of AD mice while increasing IFN-γ levels.

## 3. Conclusions

Firstly, using pseudo-ternary phase diagrams and the Box–Behnken design-effect surface method, ME formulations are screened and prepared to obtain O/W type PO-ME with a particle size of 24.83 nm, which can improve the solubility and stability of PO. PO-ME and shows high stability after undergoing centrifugation and freeze–thaw cycles, respectively. Secondly, introducing Carbopol-940 into PO-ME can increase the viscosity of the system and prolong the residence time of the formulation on the skin surface. In vitro transdermal experiments have confirmed that PO-ME and PO-MEG can cross the skin barrier. Furthermore, the skin irritation experiment on the back of rats and cytotoxicity assay in vitro investigated the skin safety of PO-MEG, and the results showed that no irritation was observed on the back of rats, and the skin tissue remained structurally intact. This indicates that PO-MEG is safe for dermal use as a transdermal drug delivery formulation. Finally, the application of PO-MEG via transdermal administration in the treatment of AD model mice showed that PO-MEG may counteract AD by regulating the immune response of mice, and PO-MEG can effectively alleviate symptoms such as erythema, edema, and scars in AD mice. Elisa and histopathological results also showed that PO-MEG can reduce the thickening of the epidermis, increase mast cells, decrease IFN-γ, and increase IL-4 and IgE in AD mice, and has excellent therapeutic effects on inflammation. In conclusion, the local microemulsion gel delivery system has shown certain advantages in drug delivery and in restoring the stability of the internal environment of the skin.

## 4. Materials and Methods

### 4.1. Materials and Animals

Patchouli oil (PO), ethyl oleate (EO), Patchouli alcohol (purity > 98%), Pogostone (purity > 98%), castor oil ethoxylated ether-40 (EL-40), and carbopol-940 were purchased from Shanghai Yuanye Biotechnology Co., Ltd. (Shanghai, China). Anhydrous ethanol and acetone were bought from Tianjin Kemo Chemical Reagent Co., Ltd. (Tianjin, China). Sudan III and methylene blue were purchased from Sinopharm Chemical Reagent Co. (Shanghai, China). Pure water was purchased from Wahaha Group Co., Ltd. (Hangzhou, China) and Hexyl hydride (GC > 98%) was obtained from Shanghai McLean Biochemistry Science and Technology Co., Ltd. (Shanghai, China). All these used reagents and solvents were of analytical grade, except for pure water and n-hexane (chromatographic grade). Sprague Dawley (SD) rats, female, 6–8 weeks old, weighing (200 ± 20 g) and of SPF grade, were obtained from Inner Mongolia Medical University Animal Center (SCXK: 2016-0001). BALB/c mice (female), 6 weeks old, were purchased from Beijing Spefo Laboratory Animal Co. (SCXK: 2024-0001) (Beijing, China). Abcell Biology (Beijing, China) provided the HaCaT cell lines. Ruixin biology (Quanzhou, China) provided the IL-4, IFN-γ, and IgE ELISA kits. The Cell Counting Kit-8(CCK-8) was obtained from Meilun biology (Dalian, China).

### 4.2. Establishment of GC-MS Chromatographic Analysis Method

Gas chromatographic conditions: An HP-5MS capillary quartz column was used with helium (He) as the carrier gas at a constant flow rate of 10.0 mL/min. The injection volume was 1.0 μL. The inlet port was in shunt mode with an initial temperature of 180 °C, held for 10 min, followed by an increase to 230 °C at a rate of 5 °C per minute, and held for 3 min. The detector temperature was set to 280 °C with a split ratio of 10∶1. The inlet port temperature was maintained at 230 °C.

Mass spectrometry conditions: The ion source was an EI ion source at 230 °C, with triple quadrupole detector at 150 °C. The scanning range was *m*/*z* 20–300 amu.

### 4.3. Establishment of Content Determination Methods

Patchouli alcohol at a concentration of 1.406 mg/mL and pogostone at a concentration of 0.892 mg/mL were used as the control master batch. A test solution was prepared by dissolving 0.01 g of PO in 10 mL of Hexyl hydride. A blank solvent sample (Hexyl hydride), the control solution, and the test solution were injected into the sample according to the chromatographic conditions specified in Section 2.2. The concentrations of patchouli alcohol were 35.15 μg/mL, 70.3 μg/mL, 140.6 μg/mL, 281.2 μg/mL, and 562.4 μg/mL, respectively. The concentrations of pogostone in the control solution were 22.3 μg/mL, 44.6 μg/mL, 89.2 μg/mL, 178.2 μg/mL, and 356.8 μg/mL. The peak areas were determined, and linear regression curves were plotted with the concentration (X) of Patchouli alcohol and pogostone in the control solution as the horizontal coordinate and the corresponding peak area as the vertical coordinate (Y). For precision, six consecutive injections of patchouli alcohol and pogostone control solutions were made under the chromatographic conditions described in Section 2.2. The peak areas were recorded, and the relative standard deviation (RSD) values were calculated. To evaluate stability, the test solution was analyzed at room temperature over a 12 h period. Injections were performed every 2 h within this timeframe, and peak areas were recorded to calculate the relative standard deviation (RSD) values for stability. For recovery tests, known concentrations of patchouli alcohol and pogostone reference solutions (50%, 100%, and 150%) were spiked into samples. After thorough mixing, the samples were analyzed under the specified chromatographic conditions, and recovery rates and RSD values were calculated.

### 4.4. Pseudo-Ternary Phase Diagram Method for Determining Prescriptions

Ethyl oleate and patchouli oil were mixed in a 1∶1 ratio to form the oil phase. Furthermore, some researchers have indicated that EL-40 improves the efficacy of skin drug delivery [33,34]. Therefore, EL-40 was selected as the emulsifier. In preliminary experiments, PO exhibited high solubility in ethanol [35], which was selected as a co-emulsifier. Mixed emulsifiers (S_mix_) were prepared in varying proportions based on a Km ratio ranging from 2:1 to 5:1. The oil phase and S_mix_ were combined in ratios of 1:9, 2:8, 3:7, 4:6, 5:5, 6:4, 7:3, 8:2, and 9:1, and then placed on a constant temperature magnetic stirrer for thorough mixing. Ultrapure water was gradually added to the mixture, and electrical conductivity was measured. The conductivity reached its maximum when the system transitioned to an O/W microemulsion state [36].

### 4.5. Entropy Weighting Assignment

The entropy weighting method determines the weights of indicators based on the information conveyed by the measured values of each indicator. This approach comprehensively accounts for various factors to assign appropriate weights [37]. Given the number of samples (m) and the number of evaluation indicators (n), an original data matrix (X_ij_) is constructed. Since higher content is preferable and smaller particle size is advantageous, increased levels of patchouli alcohol and pogostone are considered positive indicators. Equation (1) is chosen to standardize the original data for positive indicators, while Equation (2) is chosen to standardize the original data for negative indicators (particle size). This results in the standardized data matrix (Y). Next, Equation (3) is used to calculate the information entropy (E_j_), followed by Equation (4) to calculate the objective weight of each indicator (W_ij_). Finally, Equation (5) is applied to calculate the comprehensive scores (S_j_) [38,39].

(1) $$Y=\frac{X_{ij}-\min\left(X_{i}\right)}{\max\left(X_{i}\right)-\min\left(X_{i}\right)}$$

(2) $$Y=\frac{\max(X_{i})-X_{ij}}{\max(X_{i})-\min(X_{i})}$$

(3) $$E_{j}=-\ln{\left(n\right)}^{-1}\sum_{i=1}^{n}p_{ij}\ln p_{ij}$$

(4) $$W_{j}=\frac{1-E_{j}}{n-\sum E_{j}}$$

(5) $$S_{j}=\sum_{j=1}^{m}W_{j}\times X_{ij}$$

### 4.6. Box–Behnken Design

The Box–Behnken design combined with response surface methodology was employed to optimize the formulation ratios. The independent variables are related to the mass fractions of the oil phase (A), emulsifier (B), and co-emulsifier (C). The response variables include particle size (Y_1_), the content of patchouli alcohol (Y_2_), the content of pogostone (Y_3_), and the comprehensive scores (S). Optimization was carried out using Design-Expert 13 software, generating 3D response surface plots and 2D contour plots. These visualizations are very beneficial for obtaining how these independent variables affect dependent variables, and investigating the interactions between factors and their impact on response values. The optimal formulation ratios were determined based on these analyses.

### 4.7. Validation Experiment

Based on the above response surface analysis, the optimal formulation ratio of PO–EO–EL-40–ethanol–water was obtained according to a ratio of 4.35%:4.35%:19.604%:9.483%:62.213%. To further validate the accuracy of the response surface model, adjust the formulation conditions to include 4.35% ethyl oleate and patchouli oil, 19.6% EL-40, 9.5% ethanol, and 62.2% water, resulting in the preparation of three batches of PO-ME. Particle size was measured, and the contents of patchouli alcohol and pogostone were analyzed using chromatographic methods. A comprehensive score was then calculated.

### 4.8. Preparation of PO-MEG

A 1.5% carbopol-940 solution in distilled water was allowed to dissolve overnight. Subsequently, the microemulsion was mixed with the carbomer solution until a gel-like consistency was attained.

### 4.9. Quality Evaluation of PO-ME

Place the copper grid on the hydrated film, and then add a drop of the sample diluted 200 times onto the film. Negative staining was performed by adding a small amount of 2% phosphotungstic acid dropwise onto a 200 mesh copper grid, allowing it to naturally evaporate and dry. The morphology was then observed under a transmission electron microscope (TEM, FEI Talos F200S, Thermo Technologies, Inc., Denver, CO, USA). To identify the type of microemulsion, staining was conducted by adding drops of methylene blue and Sudan III solutions to the microemulsion. The samples were then observed directly for 1 min [40]. Three batches of 1 mL patchouli oil microemulsions were selected, and their particle size and ζ potential were determined using a nanoparticle size and zeta potential analyzer (SZ-100 V2, Juna Technology Corporation, Shanghai, China) to predict the stability of the microemulsion system [41]. Three batches of ME were placed in a high-speed centrifuged set at 12,000 rpm and centrifuged for 30 min. After centrifugation, the appearance of the microemulsions was observed, and their particle sizes were measured. Finally, heating–cooling cycle tests were carried out on three batches of microemulsion by subjecting them to continuous temperature cycles between −20 °C and 25 °C for a total of 6 cycles. Following these cycles, the appearance of the microemulsion was assessed and its particle sizes were measured.

### 4.10. Quality Evaluation of PO-MEG

The diluted PO-MEG was applied to a 200-mesh copper grid, allowed to dry naturally, and subsequently examined using a transmission electron microscope. pH measurements were taken for three separate batches of the PO-MEG using a pH meter. The viscosity of the gel was assessed with a digital viscometer (NDJ-5S, Jingtian Electronic Instruments Co., Ltd., Shanghai, China). Following this, three batches of PO-MEG were subjected to thermal stress by placing them in an oven at 40 ± 2 °C for 12 h, after which they were allowed to return to room temperature. The samples were then transferred to centrifuge tubes and centrifuged at 12,000 rpm for 30 min to evaluate their appearance.

### 4.11. In Vitro Transdermal Absorption of PO-MEG

Healthy SD rats weighing 180–220 g were euthanized by cervical dislocation, and their abdominal skin was excised after shaving. The subcutaneous fat and connective tissue were removed, and the skin was positioned on the diffusion interface of a Franz diffusion cell with the dermal side facing the receiving chamber. The receiving cell was filled with a 30% ethanol–saline solution. The diffusion cell was then placed in a water bath set at 32 ± 1 °C and rotated at 300 rpm for preheating and equilibration. After 0.5 h of pre-equilibration, an equal amount of PO-MEG was applied to the exposed skin area for three parallel experiments. Samples of 3 mL were collected at 2, 4, 6, 8, 10, 12, and 24 h. After each collection, an equal volume of blank receiving solution, maintained at the same temperature, was added. The collected solutions were extracted three times with n-hexane, and the extracts were combined and analyzed for content. The cumulative permeate volume (Q_n_) was calculated using Equation (6).

(6) $$Q_{n}=\frac{V_{0}}{A}\times C_{n}+\sum_{i=1}^{n-1}\frac{V_{i}}{A}\times C_{i}$$

where C_n_ is the concentration in the nth point; V_0_ is the diffusion cell volume; C_i_ is the concentration at the time point; V_i_ is the volume of each sample; and A is the diffusion cell area.

### 4.12. Cytotoxicity Assay In Vitro

Cell viability of human skin epidermal keratinocytes (HaCaT) co-cultured with PO-MEG and Blank-G was assessed using the Cell Counting Kit-8 (CCK-8) assay. HaCaT cells in the logarithmic growth phase were seeded at 1.5 × 10^4^ cells per well in 96-well plates and cultured for 24 h to allow cell adhesion. The culture medium was then replaced with 200 µL of PO-MEG or Blank-G solution in each well, while the control group was treated with 200 µL of fresh culture medium. The cells were incubated for 24, 48 and 72 h, followed by the addition of 10 µL of CCK-8 reagent. After 1 to 1.5 h of incubation, absorbance was measured at 450 nm using a microplate reader (Infinite^®^ M Nano, Tecan, Mannedorf, Switzerland) to determine cell viability for each group.

### 4.13. Skin Irritation Study

SD rats were depilated on both sides of their shaved backs, covering an area of approximately 5 × 5 cm^2^. PO-MEG was applied to the left depilated area, while a blank substrate was applied to the right. The SD rats were randomly divided into intact skin and damaged skin groups. Visible changes on the skin surface, such as complete and edema, were monitored 24, 48, and 72 h after application preparation, both in single and continuous applications over 7 days. The skin was graded into 5 categories based on the erythema and edema caused by the irritation response. The intensity of the stimulus was then assessed according to Table 4, calculated as the mean irritation response score = (total erythema formation score + total edema formation score)/number of test animals [42]. Stimulation response intensity standards: scores 0.00~0.49: non-stimulating, 0.50~2.99: mildly stimulating, 3.00~5.99: moderately stimulating, 6.00~8.00: highly stimulating.

### 4.14. Experimental Animals and Modeling Methods

**Figure 10:**
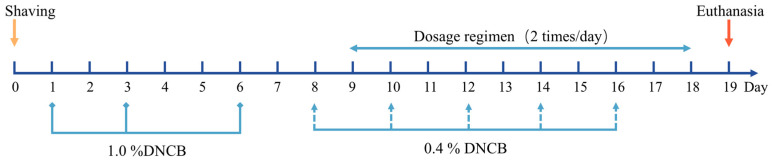
Schematic diagram of the treatment program.

Under standard laboratory conditions, mice were housed at 20 to 28 °C and 50% to 60% humidity, with a 12 h light–dark cycle and free access to food and water. All animals were acclimatized for one week before the start of the experiment. To induce the AD model, the mice were first shaved on an area of approximately 3 × 3 cm^2^ on their backs and sensitized with 100 μL of a 1% DNCB solution (acetone/olive oil, 4:1, *v*/*v*) three times (in addition to the control group). Subsequent repeated challenges with 100 μL of a 0.4% DNCB solution five times induced AD-like skin lesions such as erythema, skin thickening, desquamation, oozing blood, and crusting [43,44,45]. The experiments included control, Positive control (DNCB), free PO(PO), not nanoscaled patchouli oil gel (PO-MG), PO-MEG, blank gel (Blank-G), and Desonide groups, each consisting of 12 mice. Desonide, which has been clinically used for AD treatment, served as a positive control. To ensure effective drug absorption, all treatments were administered topically for 10 days twice daily [46]. A schematic diagram of the treatment protocol is depicted in Figure 10.

### 4.15. Scoring of Skin Lesions

The severity of lesions on the back skin of rats was scored on days 2, 7, 9, 13, and 18. Clinical skin lesion scores based on erythema, desquamation, vesiculation, and crusting were used to evaluate the extent of skin lesions in mice. Each symptom was evaluated as follows: 0 = none, 1 = mild, 2 = moderate, and 3 = severe [47], resulting in a maximum total score of 12. The total skin lesion score was determined by summing all individual dermatitis scores.

### 4.16. Histopathological Analysis

The dorsal skin was obtained from rats after euthanasia. The dorsal skin was then fixed in 4% paraformaldehyde for 24 h. The dorsal skin was embedded in paraffin, stained with hematoxylin-eosin (H&E), and subsequently stained with toluidine blue to observe epidermal lesions and count dermal mast cells. Four consecutive microscopic fields (100× magnification) were randomly selected to measure the epidermal thickness of rat skin (scale bar = 400 μm). Simultaneously, mast cells were counted in four consecutive microscope fields per 0.25 mm^2^ at 400× magnification. All histopathological analyses were performed using a light microscope.

### 4.17. Enzyme-Linked Immunosorbent Assay

After cervical dislocation of the mice, the back skin tissue was obtained from the rats and appropriately sheared with scissors before being homogenized using tissue homogenization (P23, ldar-Oberstein, Germany) for 1 h. The skin tissue homogenate was collected, and the levels of IL-4, IFN-γ, and IgE were quantified using enzyme-linked immunosorbent assay (ELISA) kits according to the manufacturer’s instructions.

### 4.18. Statistical Analysis

All data expressed as mean ± standard deviation (SD). Statistical significance was determined using one-way ANOVA and Dunnett’s *t*-test for log-transformed data, with significance set at *p* ≤ 0.05. Compared with the control group, ### *p* < 0.001 and compared with the DNCB group, * *p* < 0.05, ** *p* < 0.01, *** *p* < 0.001.

## Figures and Tables

**Table 1:** Box–Behnken experimental design table with response results (n = 3).

A	B	C	Y_1_ (nm)	Y_2_ (mg/mL)	Y_3_ (mg/mL)	Score
5.6	14	10.35	25.167	18.781	2.927	0.349
5.6	17.34	6.92	30.767	25.696	3.002	0.475
2.5	14	6.92	17.467	12.011	2.636	0.115
2.5	17.34	10.35	16.330	12.417	2.613	0.115
2.5	20.69	6.92	15.067	13.352	2.670	0.168
8.7	20.69	6.92	24.367	36.238	3.210	0.796
5.6	20.69	10.35	19.266	31.148	2.597	0.395
5.6	17.34	6.92	27.200	31.811	3.472	0.856
8.7	17.34	10.35	27.000	31.227	3.49	0.857
5.6	17.34	6.92	27.633	30.002	3.205	0.675
5.6	17.34	6.92	24.433	31.727	3.067	0.642
8.7	14	6.92	33.667	24.039	3.389	0.65
5.6	17.34	6.92	25.733	30.148	3.327	0.755
8.7	17.34	3.5	28.433	25.614	3.241	0.619
5.6	14	3.5	22.667	26.362	2.586	0.294
5.6	20.69	3.5	19.133	20.897	3.014	0.463
2.5	17.34	3.5	15.733	11.745	2.746	0.181

**Table 2:** Comparison of cumulative transdermal osmotic parameters of PO-MEG groups (n = 3).

Groups	Cumulative Permeability (µg/cm^2^)
PO-MEG group (Patchouli alcohol)	136.94 ± 6.58
PO-MEG group (Pogostone)	36.93 ± 1.4

**Table 3:** Evaluation results of skin irritation intensity.

Group		Complete Skin			Damaged Skin		
		24 h	48 h	72 h	24 h	48 h	72 h
Single-dose administration	PO-MEG	0	0	0	0.14	0.14	0
	Blank-G	0	0	0	0.14	0	0
Multiple drug delivery	PO-MEG	0	0	0	0	0	0
	Blank-G	0	0	0	0	0	0
Single-dose administration	PO-MEG	0	0	0	0	0	0
	Blank-G	0	0	0	0	0	0
Multiple drug delivery	PO-MEG	0	0	0	0.28	0.28	0
	Blank-G	0	0	0	0.28	0	0

**Table 4:** Scoring criteria of the ear skin injury in rats.

Grading Project	Score	Reaction Strength	Grading Project	Score	Reaction Strength
Erythema	0	No erythema	Edema	0	No edema
	1	Slight erythema		1	Slight edema
	2	Moderate erythema		2	Moderate edema
	3	Severe erythema		3	Severe edema
	4	Severe erythema and Eschar skin		4	Severe edema and expanded in scope

## Data Availability

The data presented in this study are available on request from the corresponding author.
